# Variation of Arbuscular Mycorrhizal Fungi Communities Between Root and Rhizosphere Soil of Endangered Plant *Heptacodium miconioides* Along Elevation Gradient

**DOI:** 10.3390/jof11030222

**Published:** 2025-03-14

**Authors:** Yueling Li, Guangyu Luo, Shijie Wu, Dang Yang, Pengpeng Lv, Zexin Jin, Junmin Li

**Affiliations:** 1 Zhejiang Key Laboratory for Restoration of Damaged Coastal Ecosystems, School of Life Sciences, Taizhou University, Taizhou 318000, China; liyl@tzc.edu.cn (Y.L.); luogy@tzc.edu.cn (G.L.); wushijietzc@163.com (S.W.); yangdangyd@163.com (D.Y.); lvpeng0903@163.com (P.L.); 2 Institute of Ecology, School of Life Sciences, Taizhou University, Taizhou 318000, China

**Keywords:** arbuscular mycorrhizal fungi, endangered plant, elevation gradient, diversity, root and rhizosphere soil

## Abstract

Arbuscular mycorrhizal fungi (AMF) are considered crucial for the survival of many endangered plant species. However, the dynamics of AMF communities in the roots and rhizosphere soil of *Heptacodium miconioides*, particularly along elevation gradients, remain underexplored. This study investigates AMF colonization, spore density, and community structure in the root and rhizosphere soil of *H. miconioides* across an elevation range from 306 to 1028 m a.s.l., employing high-throughput sequencing. Our results show that AMF colonization and spore density in *H. miconioides* increased with elevation. *Glomus* was the dominant genus in both root and rhizosphere samples. Elevation significantly influenced the AMF community structure and diversity in the root, with alpha diversity decreasing linearly with elevation. In contrast, no significant elevation-related changes were observed in the rhizosphere soil alpha diversity. The difference in AMF beta diversity between the root and rhizosphere soil was lowest at the highest elevation. Compared to the rhizosphere soil, the degree and degree centralization of AMF community co-occurrence networks in the root showed a significant increase at higher elevations. Variations in soil properties, particularly soil pH, available phosphorus, and total nitrogen levels strongly influenced AMF communities in rhizosphere soil, while nitrate nitrogen, available potassium, and acid phosphatase were correlated with AMF communities in the root. These findings highlight the impact of elevation on AMF communities in both root and rhizosphere soil, providing valuable insights for the habitat restoration and conservation efforts for this species.

## 1. Introduction

Endangered plant species are vital components of natural ecosystems and global biodiversity [1]. However, these species are increasingly threatened by factors such as over-exploitation, habitat loss, invasive species, and environmental changes, including global warming and acid rain [2]. As a result, protecting endangered plants and enhancing the viability of their populations has become a critical conservation priority. Soil microorganisms are required to maintain the stability of an ecosystem [3], with arbuscular mycorrhizal fungi (AMF) being key symbionts for over 80% of terrestrial plants. AMF serve as a crucial link between aboveground and belowground ecosystems [4]. These fungi enhance mineral nutrient uptake, improve soil stability, and increase plant resilience to environmental stressors [5], while also influencing plant fitness and reproduction [6]. Recently, AMF’s role in the conservation of endangered plants has gained increasing attention. Numerous studies have demonstrated that rare and endangered plant species exhibit improved performance and greater mycorrhizal dependence in the presence of AMF [7,8]. For example, inoculating the endangered species *Pterocarpus santalinus* with AMF symbionts promoted seedling growth and facilitated successful field establishment [9]. Likewise, Wang et al. [10] found that AMF inoculation helped protect *Zelkova serrata* from acid rain stress by enhancing photosynthetic capacity, osmolyte regulation, and activating antioxidant enzymes. These findings underscore the potential of AMF to support the survival and environmental resilience of endangered plants, thereby offering a promising approach to their conservation.

Mountain elevation gradients, with their varying environmental conditions, provide a useful model for studying soil microbial patterns, including AMF [11]. Elevation induces distinct changes in climate, seasonality, vegetation, and soil properties, all of which influence microbial communities [12]. While the relationship between AMF abundance and diversity along elevation gradients has been widely studied, the results remain inconclusive. For instance, some studies have reported a negative correlation between AMF colonization, spore density, and community diversity with elevation in Tibetan alpine grasslands [13,14]. In contrast, Vieira et al. [15] observed a higher Shannon diversity of AMF species at mid-to-high elevations in tropical mountains. Furthermore, Yu et al. [16] found that elevation positively correlated with the relative abundances of *Ambispora* and *Glomus* in the rhizosphere of *Siraitia grosvenorii*, significantly shaping AMF community composition. Other studies, such as Zhang et al. [17], suggested a bimodal pattern of AMF diversity with increasing elevation in Mt. Taibai, Qinling Mountains. These disparate findings highlight the need for further research to establish a comprehensive understanding of how AMF diversity responds to elevation in natural ecosystems.

AMF in both the root and rhizosphere soil play distinct yet complementary roles in supporting plant growth and health. Previous studies have shown that functional groups of AMF in these two environments differ significantly in terms of biomass allocation [18]. For example, *Rhizophagus* species tend to allocate more biomass to the root, while *Acaulospora* species are more likely to allocate biomass to the rhizosphere soil. This differentiation suggests that AMF in the roots are specialized for nutrient exchange and stress tolerance, whereas AMF in the rhizosphere soil are more diverse, contributing to nutrient acquisition, soil structure, and microbial interactions [19,20]. Stevens et al. [21] demonstrated that the soil AMF community structure is primarily influenced by environmental factors, while root AMF communities are more strongly impacted by disturbances and host–plant interactions. Forest management practices, such as the mixed forest management of *Juglans mandshurica*, were reported to alter the AMF composition in the soil but had minimal effect on the root AMF community [22]. These findings suggest that the AMF species in roots are different from those in the rhizosphere soil. However, most studies to date were mainly carried out on farmland [23] and grassland ecosystems [24], with limited research on AMF diversity changes in both root and rhizosphere soils in mountainous ecosystems. Studying AMF distribution patterns along elevation gradients can provide valuable insights into the factors driving elevation-related changes in diversity.

*Heptacodium miconioides* Rehd., a member of the monotypic genus *Heptacodium* in the Caprifoliaceae family, is an endemic and endangered deciduous tree. As a perennial species, *H. miconioides* occupies a unique position in the evolutionary history of Caprifoliaceae and holds significant scientific and ornamental value [25]. However, due to habitat fragmentation, low reproductive rates, poor environmental conditions, and human activities the natural populations of *H. miconioides* have been severely threatened [26]. It is classified as a second-class national protected plant in China and is listed as a vulnerable species on the IUCN Red List [27,28]. Previous studies have shown that *H. miconioides* forms a beneficial symbiotic relationship with AMF [29]. For instance, inoculation with *Rhizophagus intraradices* has been shown to enhance drought tolerance and promote seedling growth in *H. miconioides* [28]. Here, we used root and rhizosphere soil samples from *H. miconioides* across five elevation gradients in Dongbai Mountain to perform Illumina Miseq sequencing and analyze AMF diversity changes and community composition. The objective of this work was to achieve a deeper understanding of the diversity and structure of AMF communities in the root and rhizosphere soil associated with *H. miconioides* along elevation gradients. We aimed to: (1) compare the AMF community composition between the root and rhizosphere soil; (2) evaluate the response of AMF community diversity and distribution patterns to elevation gradients; and (3) assess the influence of edaphic factors and soil enzyme activity on AMF community structure in both the root and rhizosphere soils. Our results could provide insights into the role of AMF in the conservation, growth, and habitat restoration of *H. miconioides* and other endangered plant species.

## 2. Materials and Methods

### 2.1. Study Area

**Figure 1:**
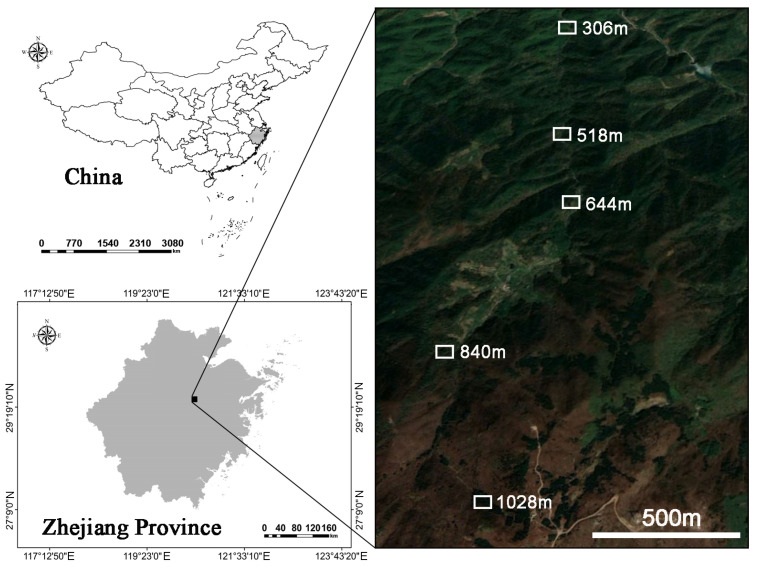
The location of the study area of the Heptacodium miconioides population on Dongbai Mountain, Zhuji City, Zhejiang Province, China.

The study was conducted in Dongbai Mountain (29°30′42.11″ N, 120°26′12.57″ E), located in Zhuji City, Zhejiang Province, China (Figure 1). The region has a subtropical monsoon climate, characterized by an average annual temperature of 11 °C, annual rainfall exceeding 1500 mm, with extreme temperatures ranging from −10.7 °C to 32 °C. The frost-free period lasts approximately 180 days. The highest elevation on Dongbai Mountain is 1194.6 m, which is one of the primary habitats for *H. miconioides*. The dominant soil type is hilly mountain red loam.

### 2.2. Root and Rhizosphere Soil Sampling

In mid-July 2019, five elevation gradients (306, 518, 644, 840, and 1028 m) were selected for sampling along the mountain from its base to summit. At each elevation, four independent replicate plots (5 m × 5 m) were randomly established, with two healthy *H. miconioides* individuals selected per plot, ensuring a minimum distance of 3.5 m between adjacent plots. Fine roots (<2 mm diameter) were collected from the 0–10 cm depth, and rhizosphere soil was gently brushed off using a soft-bristled paintbrush. Root and rhizosphere soil from two trees within each plot were combined into a single sample, yielding a total of 20 samples. Rhizosphere soil was passed through a 5 mm sieve to remove root debris and small stones. The root and rhizosphere samples were processed as follows: one portion of the root was preserved in FAA solution for AMF colonization analysis; a second portion of the rhizosphere soil was air-dried for physicochemical properties and enzyme activity assays; and the remaining root and rhizosphere soil was stored in ice boxes and subsequently kept at −80 °C for DNA extraction.

### 2.3. Soil Properties and Analysis

The pH of soil (soil–water = 1:2.5 *w/v*) was measured with a calibrated pH meter. The contents of soil water (WC) and soil organic matter (OM) were determined via the drying method and the K_2_Cr_2_O_7_/FeSO_4_ method, respectively [30]. Total nitrogen (TN) and total phosphorus (TP) were measured using a continuous flow analyzer (AA3, Germany) as previously described [31]. Ammonium nitrogen (NH_4_^+^-N) and nitrate nitrogen (NO_3_^−^-N) were extracted with KCl solution and analyzed using the AA3 continuous flow analyzer. Available phosphorus (AP) was extracted with NaHCO_3_ solution and measured with the same analyzer, while available potassium (AK) was measured using inductively coupled plasma optical emission spectrometry (ICP/OES, Optima 2100DV, Shelton, CT, USA). Soil enzyme activities were measured as follows: sucrase activity was determined by the 3,5-dinitrosalicylic acid colorimetric method, and catalase and acid phosphatase activities were measured according to the published report [32].

### 2.4. AMF Colonization and Spore Density

AMF colonization was assessed by collecting fine roots (<1 mm diameter) of *H. miconioides* and preserving them in FAA solution (formalin: glacial acetic acid: 50% ethanol = 5:5:90) at 4 °C. AMF colonization rates for hyphae, vesicles, and arbuscules in the roots were determined using acid fuchsin staining [33], with data extracted via an Axio Scope A1 light microscopy (Zeiss, Jena, Germany). For each biological replicate, 30 root segments of approximately 1 cm in length were randomly selected and loaded onto glass slides, and then fixed with a lactic acid/glycerol mixture (*v*/*v* = 1:1). Colonization rates were quantified by the gridline intersect method [34]. AMF spores were extracted from 10 g of air-dried soil samples after 20-, 100-, and 300-mesh wet sieving and 60% sucrose centrifugation [35]. Spore density was counted under a binocular stereomicroscope with gridline method.

### 2.5. DNA Extraction, MiSeq Sequencing, and Bioinformatic Analysis

Total microbial genomic DNA was extracted and purified from all rhizosphere soil and root samples using an E.Z.N.A.^®^ Soil DNA Kit (Omega, Auburn, WA, USA). DNA quality was checked in 1% agarose gel electrophoresis and its concentration was assessed using a NanoDrop2000 system (Thermo Scientific, Waltham, MA, USA). The fungal 18S rRNA gene was amplified with the primers AMV4.5NF (5′-AAGCTCGTAGTTGAATTTCG-3′) and AMDGR (5′-CCCAACTATCCCTATTAATCAT-3′) [36]. The PCR procedure and conditions were carried out according to the method of Ji et al. [22]. The PCR products were separated by 2% agarose gels, stained with ethidium bromide, and visualized under a UV transilluminator apparatus (Bio-Rad, Hercules, CA, USA). The extracted PCR products were then purified using a PCR Clean-Up Kit (YuHua, Shanghai, China), and quantified using Qubit 4.0 (Thermo Fisher, Waltham, MA, USA). Purified amplicons were pooled in equimolar amounts and sequenced in paired-end mode on an Illumina Nextseq2000 platform (Illumina, San Diego, CA, USA).

Raw sequencing reads were de-multiplexed using an in-house Perl script, quality-filtered with fastp (v0.19.6) [37], and merged with FLASH (v1.2.7) [38]. The resulting optimized sequences were clustered into operational taxonomic units (OTUs) at a 97% sequence similarity threshold using UPARSE 7.1 [39]. Taxonomic assignments for each OTU representative sequence were performed using the RDP Classifier (v2.2) against the maarjam20220506/AM species database with a confidence threshold of 0.7 [40].

### 2.6. Statistical Analysis

The effects of elevation on soil characteristics were evaluated using one-way ANOVA, followed by post hoc comparisons with either LSD or Dunnett’s T3 tests at a 5% significance level, conducted using SPSS software (IBM, Armonk, NY, USA, v22.0). Linear regression analysis was performed to assess the relationship between AMF colonization rates, spore density, and AMF community alpha diversity in the root and rhizosphere soils of *H. miconioides* across the elevation gradient.

For the Illumina MiSeq sequencing data, rarefaction curves and alpha diversity indices, including the number of observed OTUs, Chao1, and Shannon indexes, were generated using Mothur v1.30.1 [41]. The common and unique OTUs across different samples were identified using Venn diagrams, generated with R software (v3.3.1). To visualize the composition of the AMF community at the genus level, a community bar plot was created using R (v3.3.1). Differences in alpha diversity, including Chao1 and Shannon indices, between the root and rhizosphere soils were analyzed using Student’s *t*-tests at the 5% significance level.

The similarity of AMF communities across different samples was evaluated using non-metric multidimensional scaling (NMDS), based on Bray–Curtis dissimilarity, in R (v2.5-3). Differences in AMF alpha diversity (Shannon index and Chao1 index) and beta diversity (NMDS1 and NMDS2) across the elevation gradient were calculated according to the formula described by Zhao et al. [42]. Statistical significance was assessed using the Adonis test.

The co-occurrence networks were constructed to explore the internal AMF community relationships across the samples at different elevations. A correlation between two nodes was considered to be statistically robust (Spearman’s correlation coefficient over 0.7 or less than −0.7) and significantly correlated (*p*-value less than 0.01). Network topology parameters (degree and degree centralization) were calculated, and the networks were visualized using Networkx (v.1.11).

Canonical correlation analysis (CCA) was conducted to examine the relationship between environmental factors and the AMF community using R (v2.4.3). The Mantel test, with a Monte Carlo simulation (999 randomizations), was employed to assess the correlation between the Euclidean distances of environmental variables and the AMF community. Pearson correlation analysis was used to explore the relationships between soil properties and AMF colonization rate, spore density, Shannon and Chao1 diversity indices, and the AMF community composition (genus level) in both the root and rhizosphere soils.

## 3. Results

### 3.1. Soil Characteristics Along the Elevation Gradient

The variations in soil environmental factors and enzyme activities along the elevation gradient are summarized in Table 1. As elevation increased, there were significant positive correlations with soil AK (*R*^2^ = 0.31, *p* = 0.006), OM (*R*^2^ = 0.85, *p* < 0.001), WC (*R*^2^ = 0.44, *p* < 0.001), catalase (*R*^2^ = 0.27, *p* = 0.012), and acid phosphatase (*R*^2^ = 0.32, *p* = 0.006), while soil pH significantly decreased (*R*^2^ = 0.63, *p* < 0.001). The NO_3_⁻-N content (*R*^2^ = 0.57, *p* < 0.001) initially decreased and then increased with elevation. However, no significant differences were observed in the AP, TP, TN, NH_4_⁺-N, or sucrase levels across elevations.

### 3.2. AMF Colonization and Spore Density Along the Elevation Gradient

**Figure 2:**
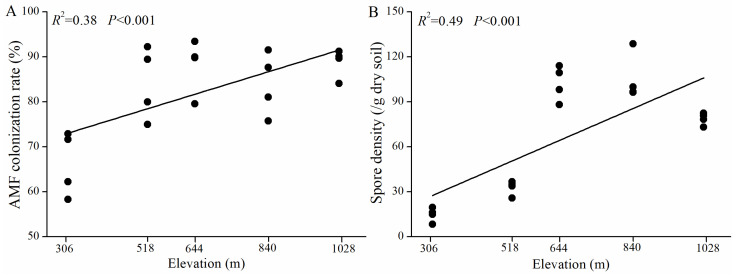
Linear correlations between elevation and AMF colonization rate (**A**) and spore density (**B**) of *H. miconioides*.

The roots of *H. miconioides* exhibited robust AMF colonization, with colonization rates ranging from 58.33% to 93.44% (Figure 2). Both the AMF colonization rate (*R*^2^ = 0.38, *p* < 0.001) and spore density (*R*^2^ = 0.49, *p* < 0.001) showed significant positive correlations with elevation. The Pearson correlation analysis revealed that AMF colonization was positively correlated with WC and catalase but negatively correlated with pH (Table S1). Spore density was positively correlated with WC, TN, catalase, and acid phosphatase, and negatively correlated with pH.

### 3.3. Overall Sequencing and Taxonomic Assignments in Root and Rhizosphere Soil

**Figure 3:**
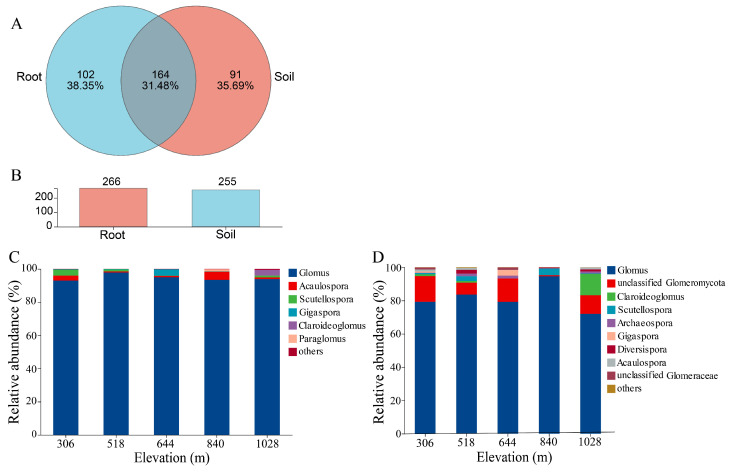
Relative abundance of AMF community composition in root and rhizosphere soil of *H. miconioides* along elevation gradients. (**A**) Venn diagram showing number of common and unique OTUs between root and rhizosphere soil. (**B**) Bar graphs representing total number of OTUs between root and rhizosphere soil. Relative abundance of AMF community composition at genus level in root (**C**) and rhizosphere soil (**D**).

The sequencing generated a total of 476,816 reads (range: 22,985–24,634) from the root samples and 458,501 reads (range: 14,888–24,656) from the rhizosphere soil samples (Table S2). Based on the maarjam20220506/AM species database, 476,816 reads were classified into one phylum, four classes, six orders, twelve families, thirteen genera, and eighty-eight species in the root samples. In the rhizosphere soil, the data revealed one phylum, four classes, six orders, eleven families, thirteen genera, and seventy-six species. These sequences were grouped into 266 and 255 AMF OTUs in the root and rhizosphere samples, respectively, based on 97% sequence similarity, with 164 OTUs shared between the two environments (Figure 3A,B). Rarefaction curves for alpha diversity (Figure S1) showed a leveling off, indicating an adequate sequencing depth and suggesting that AMF diversity in the roots was higher than that in the rhizosphere soil.

At the genus level (Figure 3C,D), five genera were identified in the roots, including *Glomus*, *Scutellospora*, *Acaulospora*, *Gigaspora*, and *Claroideoglomus*. *Glomus* (94.57%) was the most abundant genus across all elevation gradients, followed by *Acaulospora* (2.08%). The genus-level analysis revealed that *Glomus*, *Scutellospora*, and *Acaulospora* were consistently present at all elevations, indicating their widespread distribution in Dongbai Mountain. In contrast, *Paraglomus* and *Claroideoglomus* were exclusively found in high-elevation root samples. In the rhizosphere soil, nine genera were detected, including *Glomus* (81.74%), unclassified *Glomeromycota* (9.68%), *Claroideoglomus* (2.98%), *Scutellospora* (1.61%), *Archaeospora* (0.98%), *Diversispora* (0.85%), *Gigaspora* (0.83%), *Acaulospora* (0.72%), and unclassified *Glomeraceae* (0.55%). Notably, *Claroideoglomus* exhibited significant differences (*p* = 0.043) across rhizosphere samples (Figure 3C,D, Table S3). A more detailed analysis at 1028 m elevation revealed the significant dominance of *Glomus* in both the root and rhizosphere soils (Figure S3). While *Scutellospora*, *Acaulospora*, *Gigaspora*, and *Claroideoglomus* were detected in samples of both the root and rhizosphere, unclassified *Glomeromycota*, *Archaeospora*, *Diversispora,* and unclassified *Glomeraceae* were exclusive to the rhizosphere soil.

### 3.4. Alpha Diversity of AMF in Root and Rhizosphere Soil

**Figure 4:**
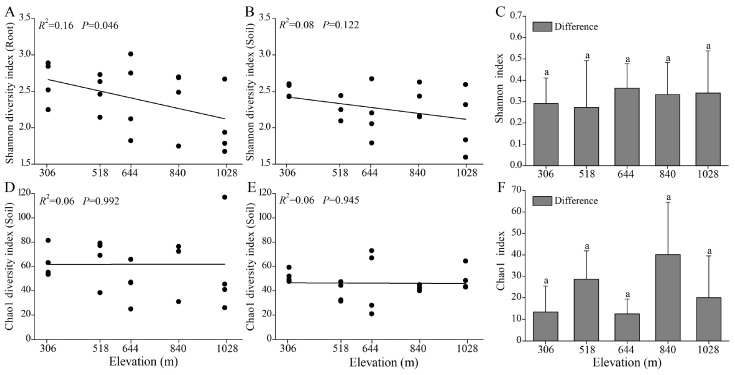
Linear correlations between elevation and alpha diversity indices (Shannon and Chao1) in root (**A**,**D**), rhizosphere soil (**B**,**E**), and their differences (**C**,**F**) in *H. miconioides*. Data are presented as means ± SD. Significant differences are denoted by lowercase letters (*p* < 0.05).

Alpha diversity indices (Shannon and Chao1) were used to assess the diversity of AMF communities in the root and rhizosphere soil of *H. miconioides* (Figure 4). The Shannon index for the root AMF communities significantly declined with elevation (*R*^2^ = 0.16, *p* = 0.046), whereas the Chao1 index showed no significant correlation with elevation. Nevertheless, the Chao1 index in the roots was significantly higher than in the rhizosphere soil (*p* = 0.022; Figure S2). In the rhizosphere soil, neither the Shannon nor Chao1 indices exhibited significant changes along the elevation gradient. Additionally, no significant differences in AMF alpha diversity between the root and rhizosphere soil were observed across elevation gradients (Figure 4C,F).

### 3.5. Beta Diversity of AMF in Root and Rhizosphere Soil

**Figure 5:**
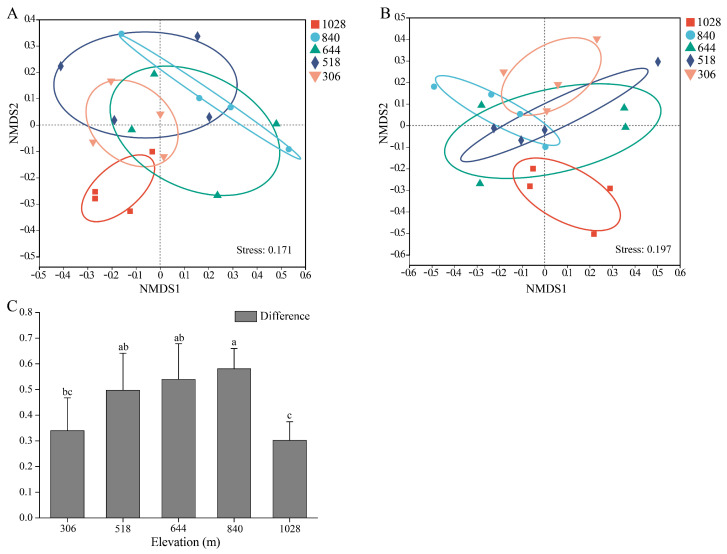
Nonmetric multidimensional scaling (NMDS) ordinations of AMF community compositions (Bray–Curtis) in root (**A**), rhizosphere soil (**B**), and their differences (**C**) based on OTU abundance along elevation gradients. Data are presented as means ± SD. Significant differences are denoted by lowercase letters (*p* < 0.05).

The NMDS analysis based on Bray–Curtis distances and Adonis tests at the OTU level revealed significant differences in the AMF community structure along elevation gradients in both the root (*R*^2^ = 0.32; *p* = 0.015) and rhizosphere soil samples (*R*^2^ = 0.26; *p* = 0.044) (Figure 5A,B). The AMF communities at the 1028 m elevation were distinctly separated from those at 840 m and 518 m. In the root samples, significant species differences were also observed between 840 m and 306 m elevations. In the rhizosphere soil, the 1028 m elevation community significantly differed from the 306 m elevation community. Interestingly, differences in AMF beta diversity between the root and rhizosphere soil initially increased and then decreased with elevation (Figure 5C). These results clearly distinguish between AMF communities in higher and lower elevations.

### 3.6. Co-Occurrence Network Analysis of AMF Community in Root and Rhizosphere Soil

**Figure 6:**
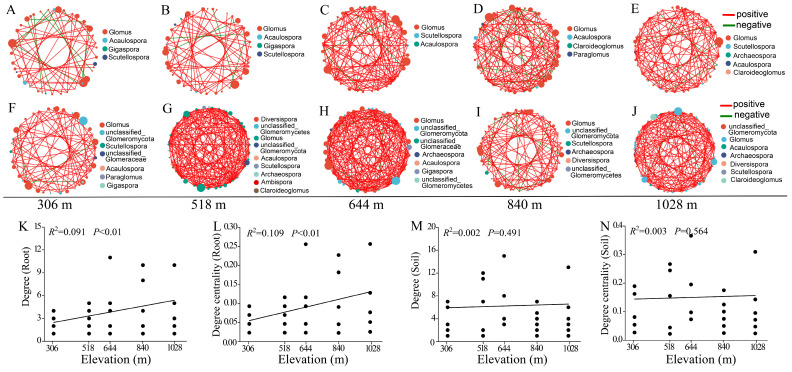
The co-occurrence networks analysis of AMF community along elevation gradients. The changes in the AMF community co-occurrence networks in the root at 306 m (**A**), 518 m (**B**), 644 m (**C**), 840 m (**D**), and 1028 m (**E**) elevation. The changes in the AMF community co-occurrence networks in the rhizosphere soil at 306 m (**F**), 518 m (**G**), 644 m (**H**), 840 m (**I**), and 1028 m (**J**) elevation. Linear correlations between elevation and topological parameters of the AMF community co-occurrence networks in the root (**K**,**L**) and rhizosphere soil (**M**,**N**) of *H. miconioides*. The positive and negative correlations are indicated by red and blue lines, respectively.

We constructed correlation networks of the AMF communities in the root and rhizosphere soil along elevation gradients (Figure 6A–J). The networks generated a total of 217 nodes (range: 40–45) connected by 424 edges (range: 51–114) from the root samples and 210 nodes (range: 38–46) connected by 657 edges (range: 69–184) from the rhizosphere soil samples (Table S4). The rhizosphere soil contained more edges than the root. In the root and rhizosphere soil co-occurrence networks, the average proportion of positive edges was 0.91 (range: 0.80–0.97) and 0.97 (range: 0.92–1), respectively (Table S4). The number of nodes and edges of the root AMF community co-occurrence networks generally increased with increasing elevation, but not with the rhizosphere soil AMF community co-occurrence network. Furthermore, correlation analysis showed that the degree and degree centralization in the root AMF community co-occurrence network significantly increased with increasing elevation gradient (Figure 6K,L). However, no significant correlations between these topological parameters and the elevation gradient were observed in the rhizosphere soil AMF community co-occurrence network (Figure 6M,N). These results suggest that elevation gradients play more important roles in shaping the AMF community co-occurrence network structure of the root than the rhizosphere soil.

### 3.7. Relationships Between Soil Characteristics and AMF Community

**Figure 7:**
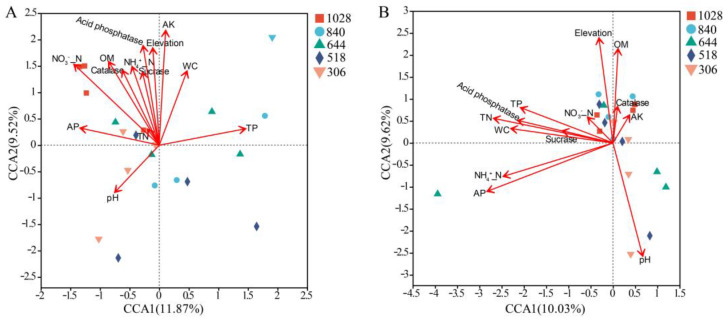
Canonical correspondence analysis (CCA) plots of AMF community compositions and soil properties in root (**A**) and rhizosphere soil (**B**) based on OTU abundance along elevation gradients.

CCA and permutest analyses identified NO₃⁻-N, AK, OM, acid phosphatase, and elevation as significant factors influencing AMF community structure in the roots (Figure 7A). In the rhizosphere soil, pH, AP, TN, and NH₄⁺-N significantly affected the AMF community composition (Figure 7B). The Mantel test results indicated that the root AMF community structure was significantly correlated with AP and TN contents, while the rhizosphere soil community was correlated with TN content (Table 2). Pearson’s correlation analysis further showed that the Shannon and Chao1 indices for AMF in the rhizosphere soil were not significantly associated with soil properties (Table S5). However, in the roots AP, TN, and catalase activity were significantly correlated with the Shannon index (Table S5).

**Figure 8:**
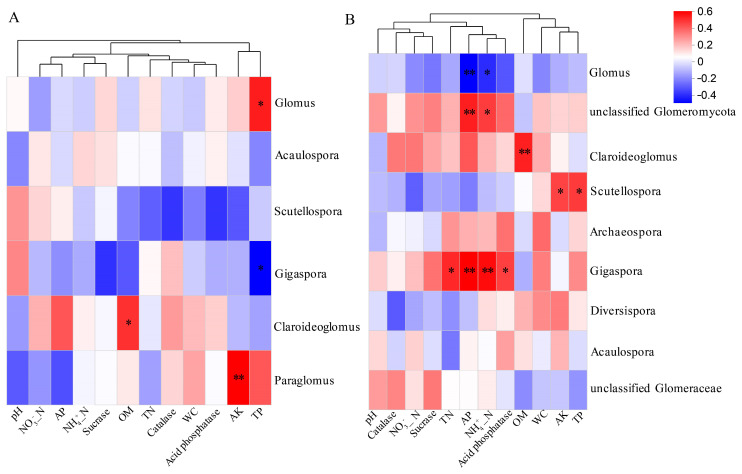
Pearson correlations between AMF communities at genus level in root (**A**) and rhizosphere soil (**B**) along elevation gradients. * *p* < 0.05; ** *p* < 0.01.

Pearson’s correlation analysis was performed to investigate the relationships between the soil properties and AMF diversity at the genus level (Figure 8). In the root samples, OM content was positively correlated with *Claroideoglomus*, while AK content showed a positive correlation with *Paraglomus* (Figure 8A). TP content exhibited a positive correlation with *Glomus* and a negative correlation with *Gigaspora*. In the rhizosphere soil, AP and NH₄⁺-N were negatively correlated with *Glomus* but positively associated with unclassified *Glomeromycota*. TN, AP, NH_4_^+^-N, and acid phosphatase were positively correlated with *Gigaspora* (Figure 8B). Furthermore, AK and TP exhibited positive correlations with *Scutellospora*.

## 4. Discussion

AMF play a crucial role in ecosystem stability and ecological restoration [4,5]. Many endangered plants form symbiotic relationships with AMF, often thriving in specialized habitats that harbor unique AMF species [43]. This study investigated the colonization and diversity of AMF in the root and rhizosphere soil of endangered *H. miconioides* across elevation gradients, identifying key environmental factors that shape AMF community structure.

### 4.1. Changes in AMF Colonization and Spore Density of H. miconioides Along Elevation Gradients

AMF colonization and spore density are critical indicators of AMF growth and their symbiotic relationships with host plants [34]. Our previous research demonstrated that AMF inoculation successfully formed a robust symbiosis with *H. miconioides* roots under greenhouse conditions, achieving a colonization rate exceeding 80% [28]. In the current study, AMF colonization and spore density increased linearly with elevation, indicating a strengthening symbiotic relationship at higher elevations. This trend aligns with the natural distribution of *H. miconioides*, which is more abundant at higher elevations. The increased AMF colonization and spore density at higher elevations likely reflect AMF’s role in enhancing soil nutrient availability and promoting plant growth. However, these findings contrast with previous studies reporting either negative correlations between AMF colonization and elevation [13,44] or non-linear trends [45]. The discrepancy may be attributed to differences in elevation ranges, as previous studies examined gradients up to 1000 m, whereas this study focused on gradients of approximately 700 m. Variations in host plant species and the harsh environmental conditions of high-altitude ecosystems may also contribute to these differences [46,47]. Additionally, spore density in the rhizosphere soil also increased linearly with elevation, corroborating the findings of Coutinho et al. [48], who reported the highest spore density at 1100 m. However, other research on *Chamaecyparis formosensis* at elevations ranging from 1200 to 2500 m found no significant elevation effect on spore density [49]. Factors such as soil nutrient availability, host dependency, natural habitat, and spore ecological adaptations likely influence AMF spore distribution patterns [14,49,50]. Pearson’s correlation analysis revealed significant positive correlations between AMF spore density and WC, TN, catalase, and acid phosphatase levels, while a negative correlation was observed with pH. AMF spore density may be linked to acidic soils, which produce higher AMF spore counts but lower taxonomic diversity compared to slightly acidic soils [51]. Our results suggest that soil moisture, TN, and enzyme activities may play important roles in enhancing soil microbial reproduction, resulting in increased mycorrhizal sporulation [52,53,54]. These findings underscore the critical influence of soil properties on the rhizosphere spore density of *H. miconioides* along elevation gradients.

### 4.2. Comparison of AMF Communities in Root and Rhizosphere Soil of H. miconioides Along Elevation Gradients

AMF occupy dual niches within the host roots and surrounding soil, playing a critical role in maintaining plant populations [43,55]. At the genus level, *Glomus* was more abundant in *H. miconioides* roots than in rhizosphere soil and consistently dominated AMF communities at all elevations. A similar dominance by *Glomus* or members of Glomeraceae has been observed in other endangered plants, including *Ulmus chenmoui* [52], *Toona ciliata* [56], and *Tetraena mongolica* [57]. The extensive colonization of *Glomus* within roots across diverse habitats enhances plant resistance and adaptability to environmental stress [58]. Nevertheless, other studies have identified Acaulosporaceae and Gigasporaceae as dominant AMF in the roots of endangered plants [59], suggesting that AMF dominance may depend on host specificity. Moreover, elevational differences also influenced the relative abundance of other AMF genera. *Paraglomus* and *Claroideoglomus* were predominantly detected at higher elevations, while *Gigaspora* was more common at lower elevations. Variations in water and nutrient availability likely shaped these distribution patterns. For instance, Gigasporaceae, known for producing extensive external hyphae, is well suited for nutrient acquisition in nutrient-poor soils [58]. The poor soil conditions at lower elevations in this study likely favored *Gigaspora*, whose well-developed external hyphae network supports nutrient absorption for host plants.

Our study also found that the AMF community compositions in roots and rhizosphere soil differed significantly, consistent with previous findings from forest ecosystems [22]. Bonfim et al. [60] reported high AMF diversity in rhizosphere soil in a Brazilian Atlantic forest, with only Glomeraceae detected within roots. These compositional differences may be due to the selective preferences of plant root systems and the varying responses of AMF taxa to root and rhizosphere environments [22]. Root-associated AMF communities are primarily shaped by plant characteristics, whereas those in rhizosphere soil are influenced by external environmental factors. Moreover, the differences in beta diversity between root and rhizosphere AMF communities initially increased along elevation gradients but decreased at the highest elevation (1028 m), resembling trends observed in Taibai Mountain alpine meadows [42]. The shift from dispersion at medium elevations (low-nutrient conditions) to aggregation at high elevations (high-nutrient conditions) indicates that abiotic factors significantly influence AMF community structure. The reduced beta diversity differences at the highest elevation suggest that environmental conditions at high elevations foster more uniform AMF community assemblies between the roots and rhizosphere soil in *H. miconioides*. Furthermore, previous studies have shown that topological parameters in co-occurrence networks, such as clustering coefficient, average degree, and degree centralization, can reflect the interaction intensity between species [61,62]. In this study, the degree and degree centralization of the root AMF community co-occurrence networks significantly increased with the increasing elevation, while no significant difference in the rhizosphere soil was observed. These findings further suggest that the interaction intensities of the AMF community within *H. miconioides* roots are more pronounced at higher elevations compared to those at lower elevations.

In addition to differences composition and distribution, alpha diversity patterns also varied between root and rhizosphere AMF communities along elevation gradients. Root AMF diversity significantly decreased with increasing elevation, aligning with previous studies reporting negative correlations between AMF diversity and elevation [13,14]. However, contrasting results from Brazilian forests, where AMF diversity increased at higher elevations [60], highlight the influence of regional environmental factors and soil characteristics. Despite the decline in root AMF diversity, no significant changes were observed in rhizosphere AMF diversity along elevation gradients. This discrepancy suggests that the reduced AMF-root symbiotic associations at high elevations are not due to the absence of symbiotic AMF in the rhizosphere but rather host plant selection against diverse fungal communities [63]. Interestingly, the Chao1 richness index for AMF communities remained stable in both root and rhizosphere samples across elevations. These findings indicate that neither the availability of AMF in rhizosphere soil nor the capacity of *H. miconioides* to establish and maintain mycorrhizal associations were significantly influenced by elevation gradients.

### 4.3. Relationship Between Environmental Factors and AMF Communities in Root and Rhizosphere Soil of H. miconioides

The structure of the AMF community in the roots and rhizosphere soil of *H. miconioides* shows distinct relationships with environmental factors. Previous studies have demonstrated that soil properties, elevation, and environmental filtering significantly influence AMF diversity, leading to spatial variations in AMF communities at local scales [55,64]. Our findings indicate that the composition of rhizosphere soil AMF communities in *H. miconioides* is strongly associated with soil pH, AP, and TN levels. Soil pH plays a crucial role in shaping AMF community structure [65], likely affecting spore formation and development [56], nutrient availability [66], and the distribution of AMF genera [67]. In this study, while pH did not significantly influence root-associated AMF communities, it had a pronounced effect on the AMF composition in rhizosphere soil, suggesting that AMF communities in the rhizosphere are more sensitive to pH changes. Additionally, AP and TN levels were significantly correlated with AMF diversity in the rhizosphere soil, aligning with previous findings [57]. Changes in AP and TN likely influence AMF spore germination, mycelial growth, and the efficiency of mycorrhizal symbiosis [68]. The Mantel test further confirmed the significant impact of TN content on AMF community structure, supporting this viewpoint.

In contrast, the root-associated AMF composition was significantly correlated with NO_3_^−^-N, AK, OM, acid phosphatase, and elevation. Correlation analysis revealed that OM content was positively associated with *Claroideoglomus*, while AK content correlated positively with *Paraglomus*. Nutrient enrichment, particularly increased OM levels, may enhance AMF reproduction, influencing community abundance and diversity [69]. AK is also known to support AMF colonization and the establishment of effective symbioses in certain plant species [70]. Acid phosphatase may have direct or indirect roles in soil and microbial-mediated phosphorus cycling, further influencing AMF abundance. Collectively, these findings suggest that variations in soil properties (pH, AP, TN, NO_3_^−^-N, AK, OM, and acid phosphatase) along elevation gradients significantly affect AMF genera, driving differences in the AMF composition between roots and rhizosphere soil. Our study underscores the ecological preferences of symbiotic AMF in response to elevation-related environmental changes within the natural distribution area of *H. miconioides*. Future research examining the influence of additional environmental factors, such as climate variables and vegetation types, could provide deeper insights into the mechanisms governing AMF community assembly and diversity along elevational gradients.

## 5. Conclusions

Our field investigation revealed a significant increase in AMF colonization rates and spore density in *H. miconioides* natural populations with the increase in the elevation gradient. The dominant AMF genera in both the root and rhizosphere soil included *Glomus*, *Claroideoglomus*, unclassified *Glomeromycota*, *Scutellospora*, *Gigaspora*, and *Acaulospora*, though their relative proportions varied with elevation. The root-associated AMF alpha diversity decreased linearly with increasing elevation, whereas the rhizosphere soil diversity remained largely unchanged. Beta diversity differences between the root and rhizosphere soil initially increased and then declined with elevation. Rhizosphere AMF communities were closely associated with soil pH, AP, and TN, while root-associated communities were significantly correlated with NO_3_^−^-N, AK, OM, acid phosphatase, and elevation. These findings provide valuable insights into the diversity and ecological preferences of the AMF species associated with *H. miconioides*. The characterization and identification of AMF species in the natural habitat of *H. miconioides* hold the potential for isolating highly effective AMF strains that have co-evolved with the species. Such strains could be harnessed as AMF inoculants to support *H. miconioides* conservation and habitat restoration efforts. These insights also offer a novel perspective for enhancing the conservation of other endangered plant species capable of forming AMF associations.

## Figures and Tables

**Table 1:** Soil physicochemical properties and enzyme activities of *H. miconioides* under different elevation gradients.

Soil Properties			Elevation			Regression
	306 m	518 m	644 m	840 m	1028 m	*R* ^2^
NH_4_^+^-N	49.14 ± 7.80 ^ab^	31.37 ± 3.19 ^c^	57.36 ± 15.76 ^a^	36.36 ± 9.31 ^bc^	54.92 ± 5.61 ^a^	0.09
NO_3_^−^-N	38.77 ± 3.42 ^b^	5.70 ± 2.73 ^d^	31.34 ± 5.61 ^bc^	24.37 ± 3.94 ^c^	51.29 ± 5.41 ^a^	0.57 **
AK	0.76 ± 0.19 ^c^	0.86 ± 0.14 ^bc^	0.88 ± 0.13 ^bc^	1.31 ± 0.33 ^a^	1.24 ± 0.29 ^ab^	0.31 **
pH	5.52 ± 0.05 ^a^	5.19 ± 0.13 ^b^	5.37 ± 0.10 ^ab^	4.86 ± 0.10 ^c^	4.91 ± 0.13 ^c^	0.63 **
OM	82.21 ± 12.1 ^c^	100.22 ± 25.92 ^c^	89.34 ± 16.24 ^c^	137.65 ± 7.83 ^b^	206.55 ± 13.31 ^a^	0.85 **
WC	8.96 ± 3.47 ^c^	17.93 ± 1.82 ^bc^	28.58 ± 9.07 ^ab^	24.17 ± 7.88 ^ab^	33.85 ± 10.34 ^a^	0.44 **
AP	11.83 ± 0.37 ^a^	9.29 ± 0.83 ^a^	10.85 ± 4.60 ^a^	9.52 ± 1.80 ^a^	12.25 ± 2.01 ^a^	0.02
TN	1.22 ± 0.07 ^b^	1.61 ± 0.49 ^b^	2.52 ± 0.56 ^a^	1.61 ± 0.57 ^b^	2.02 ± 0.45 ^ab^	0.12
TP	0.25 ± 0.03 ^b^	0.32 ± 0.02 ^ab^	0.27 ± 0.09 ^ab^	0.36 ± 0.07 ^a^	0.28 ± 0.04 ^ab^	0.01
Catalase	14.68 ± 1.96 ^bc^	14.57 ± 2.77 ^c^	17.94 ± 1.80 ^ab^	16.88 ± 0.70 ^ac^	18.57 ± 1.5 ^a^	0.27 *
Acid phosphatase	0.03 ± 0.02 ^b^	0.03 ± 0.02 ^b^	0.11 ± 0.06 ^a^	0.08 ± 0.02 ^ab^	0.13 ± 0.06 ^a^	0.32 **
Sucrase	2.64 ± 0.62 ^a^	1.32 ± 0.31 ^b^	3.48 ± 0.87 ^a^	2.41 ± 0.47 ^ab^	3.15 ± 0.89 ^a^	0.04

NH_4_^+^-N: ammonium nitrogen; NO_3_^−^-N: nitrate nitrogen; AK: available potassium; pH: pH value; OM: organic matter; WC: water content; AP: available phosphorus; TN: total nitrogen; and TP: total phosphorus. Significant differences in same column are denoted by lowercase letters (*p* < 0.05). *R*^2^ values indicate proportion of variance explained in regression analysis. Data are presented as mean ± SD (*n* = 4). * *p* < 0.05, ** *p* < 0.01.

**Table 2:** Mantel analysis of relationship between OTU relative abundance and soil parameters.

Soil Properties	Rhizosphere Soil		Root	
	*R* ^2^	*p*	*R* ^2^	*p*
NH_4_^+^-N	0.020	0.424	0.123	0.138
NO_3_^−^-N	0.056	0.306	0.147	0.058
AK	−0.015	0.513	0.041	0.342
pH	−0.003	0.497	−0.081	0.861
OM	0.114	0.164	0.138	0.092
WC	−0.066	0.689	−0.084	0.785
AP	0.038	0.369	0.238	0.021
TN	0.233	0.029	0.177	0.036
TP	−0.090	0.775	0.001	0.497
Catalase	0.164	0.072	0.050	0.296
Acid phosphatase	0.071	0.271	0.035	0.664
Sucrase	0.132	0.150	−0.051	−0.27
Elevation	0.104	0.122	−0.009	0.534

*R*^2^ values indicate proportion of variance explained in regression analysis. *p* values indicate significance level (*p* < 0.05).

## Data Availability

The dataset was deposited in the NCBI under accession numbers PRJNA1230584 (rhizosphere soil) and PRJNA1230586 (root).

## Supplementary Materials

The following supporting information can be downloaded at https://www.mdpi.com/article/10.3390/jof11030222/s1, Table S1. Pearson correlation analysis of soil properties with AMF colonization rate and spore density; Table S2. AMF sequences and mean length of sequences in each rhizosphere soil and root samples at different elevations; Table S3. Relative abundance of AMF in the *H. miconioides* rhizosphere soil and root along elevation gradient; Table S4. The topological parameters of the AMF community co-occurrence networks in rhizosphere soil and root; Table S5. Pearson correlation analysis of soil properties with Shannon and Chao1 diversity in rhizosphere soil and root; Figure S1. AMF community dilution curves for soil at different elevations; Figure S2. Alpha diversity indices of AMF communities were compared between root (*n* = 20) and rhizosphere soil (*n* = 20) samples; Figure S3. The relative abundances of *Glomus* in the root and soil samples. Values in the bar plot are expressed as mean ± standard deviation. Asterisks indicate significant difference between treatments based on Student’s *t*-test (*p* < 0.05).
