# Supplementary material for: Variation of Arbuscular Mycorrhizal Fungi Communities Between Root and Rhizosphere Soil of Endangered Plant Heptacodium miconioides Along Elevation Gradient
Source: J Fungi (Basel). 2025 Mar 14;11(3):222. doi: 10.3390/jof11030222 (PMC11942833; doi:10.3390/jof11030222)
Supplement: Supplementary file 1 [file jof-11-00222-s001.zip › jof-3495821-supplementary.pdf]

Table S1. Pearson correlation analysis of soil properties with AMF colonization rate and spore density.

Soil properties	AMF colonization rate (%)	Spore density (g^{-1} dry soil)
NH_4^+-N	-0.04	0.28
NO_3^--N	-0.07	0.19
AK	0.25	0.39
pH	-0.52*	-0.48*
OM	0.30	0.36
WC	0.53*	0.70**
AP	-0.04	-0.05
TN	0.36	0.61**
TP	0.16	0.19
Catalase	0.46*	0.53*
Acid phosphatase	0.44	0.58**
Sucrase	0.05	0.27

NH_4^+-N: ammonium nitrogen; NO_3^--N: nitrate nitrogen; AK: available potassium; pH: pH value; OM: organic matter; WC: water content; AP: available phosphorus; TN: total nitrogen; TP: total phosphorus. Significant differences by * $P < 0.05$; ** $P < 0.01$.

Table S2. AMF sequences and mean length of sequences in each rhizosphere soil and root samples at different elevations.

Samples	Rhizosphere soil		Root	
	Sequence	Mean length	Sequence	Mean length
306m-1	14888	215.72	23842	215.51
306m-2	24482	219.33	24647	215.46
306m-3	24632	214.94	24569	215.49
306m-4	24467	215.91	23794	215.69
518m-1	23636	215.51	23486	215.71
518m-2	24781	215.91	23345	215.56
518m-3	24728	215.68	23771	215.79
518m-4	23539	218.48	24893	215.41
644m-1	24401	215.69	23648	215.08
644m-2	24656	217.83	23112	215.39
644m-3	23546	215.99	23494	215.71
644m-4	24772	215.17	24190	215.59
840m-1	23873	215.70	24634	216.18
840m-2	23454	215.43	23155	215.71
840m-3	21167	215.61	23597	215.68
840m-4	18773	215.57	23828	215.45
1028m-1	23669	215.75	24087	215.60
1028m-2	24427	217.41	22985	216.49
1028m-3	22878	217.28	24499	215.64
1028m-4	17732	217.03	23240	215.61

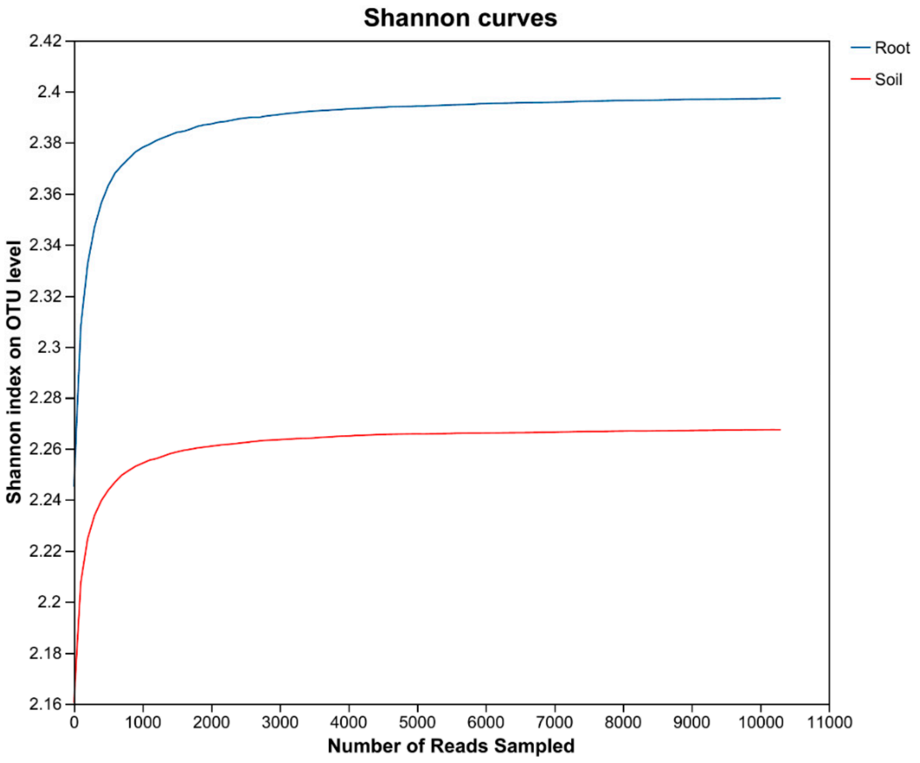

Figure S1. AMF community dilution curves for soil at different elevations.

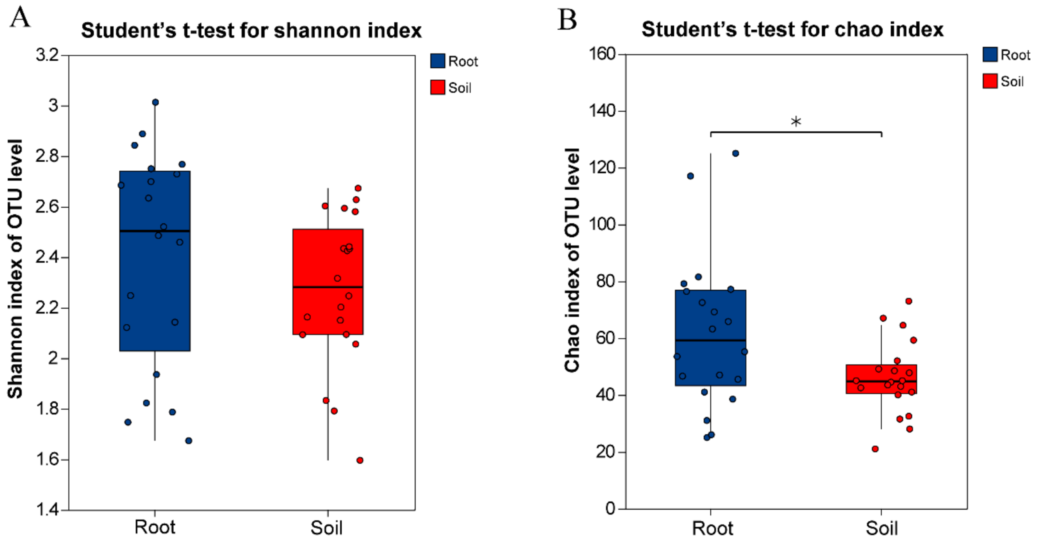

Figure S2. Alpha diversity indices of AMF communities were compared between root (n = 20) and rhizosphere soil (n = 20) samples.

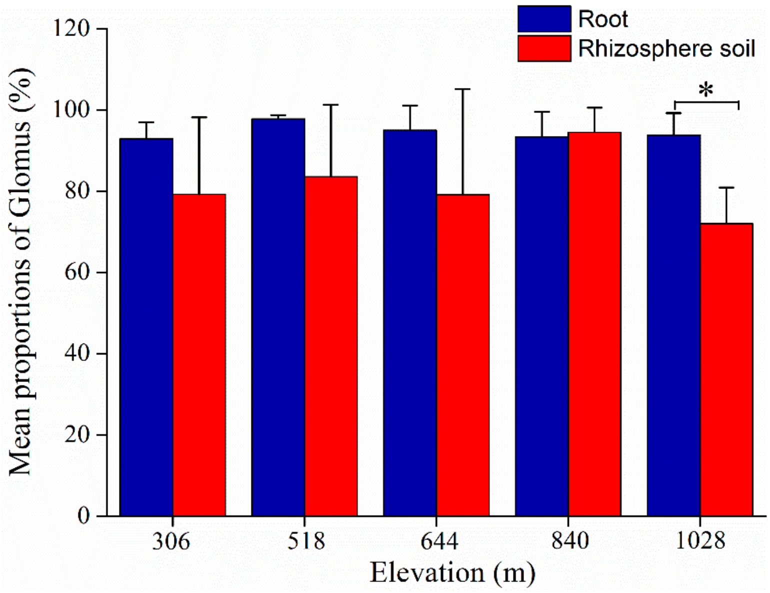

Figure S3. The relative abundances of *Glomus* in the root and soil samples. Values in the bar plot are expressed as mean ± standard deviation. Asterisks indicate significant difference between treatments based on Student's *t*-test ($P < 0.05$)

Table S3. Relative abundance of AMF in the *H. miconioides* rhizosphere soil and root along elevation gradient

**Elevation (m)**		**306**	**518**	**644**	**840**	**1028**
Rhizosphere soil	*Glomus*	79.26	83.59	79.20	94.51	72.01
	Scutellospora	0.82	2.90	0	4.01	0.30
	Acaulospora	1.09	1.17	0.05	0	1.29
	Gigaspora	0.78	0	3.38	0	0
	Claroideoglomus	1.13	0.95	0	0.02	12.80
	Unclassified_p_Glomeromycota	15.37	7.16	14.01	0.71	11.15
	unclassified_f_Glomeraceae	1.20	0	1.53	0	0
	Archaeospora	0.13	1.62	1.75	0.16	1.25
	Diversispora	0.05	2.22	0	0.51	1.13
	others	0.16	0.40	0.09	0.08	0.05
Root	*Glomus*	92.91	97.80	94.97	93.36	93.81
	Scutellospora	3.54	1.18	0.22	0.13	1.11
	Acaulospora	3.08	0.64	0.77	4.82	1.07
	Gigaspora	0.30	0.33	3.82	0.08	0
	Claroideoglomus	0	0	0	0.18	3.43
	Paraglomus	0	0	0	1.22	0
	others	0.16	0.05	0.22	0.21	0.58

Values are mean (n=4).

Table S4. The topological parameters of the AMF community co-occurrence networks in rhizosphere soil and root

**Elevation (m)**		**306**	**518**	**644**	**840**	**1028**
Rhizosphere soil	Number of nodes	38	46	42	41	43
	Number of edges	69	180	184	85	139
	Positive edges	66	180	180	78	137
	Negative edges	3	0	4	7	2
	Proportion (Positive/total)	0.96	1	0.98	0.92	0.99
	Average degree	3.63	7.83	8.76	4.15	6.47
	Degree centralization	0.10	0.17	0.21	0.10	0.15
Root	Number of nodes	44	44	44	45	40
	Number of edges	51	56	109	114	94
	Positive edges	41	52	106	99	90
	Negative edges	10	4	3	15	4
	Proportion (Positive/total)	0.80	0.93	0.97	0.87	0.96
	Average degree	2.32	2.55	4.95	5.07	4.70
	Degree centralization	0.05	0.06	0.12	0.12	0.12

Table S5. Pearson correlation analysis of soil properties with Shannon and Chao1 diversity in rhizosphere soil and root

**Soil properties**	**Rhizosphere soil**		**Root**	
	**Shannon**	**Chao1**	**Shannon**	**Chao1**
NH_4^+-N	-0.28	0.25	-0.20	-0.09
NO_3^--N	-0.25	0.30	-0.18	-0.21
AK	0.16	0.07	0.07	0.06
pH	-0.23	0.11	0.30	-0.02
OM	0.14	0.20	-0.21	-0.11
WC	0.09	0.01	-0.33	-0.06
AP	-0.34	-0.30	-0.47*	-0.24
TN	0.11	-0.12	-0.49*	-0.35
TP	-0.18	-0.24	-0.01	-0.12
Catalase	0.15	0.01	-0.51*	-0.25
Acid phosphatase	-0.01	-0.08	-0.23	-0.39
Sucrase	-0.27	-0.39	-0.35	-0.27

Significant differences by * $P < 0.05$.
